# Prediction of Incident Hypertension Within the Next Year: Prospective Study Using Statewide Electronic Health Records and Machine Learning

**DOI:** 10.2196/jmir.9268

**Published:** 2018-01-30

**Authors:** Chengyin Ye, Tianyun Fu, Shiying Hao, Yan Zhang, Oliver Wang, Bo Jin, Minjie Xia, Modi Liu, Xin Zhou, Qian Wu, Yanting Guo, Chunqing Zhu, Yu-Ming Li, Devore S Culver, Shaun T Alfreds, Frank Stearns, Karl G Sylvester, Eric Widen, Doff McElhinney, Xuefeng Ling

**Affiliations:** ^1^ Department of Health Management Hangzhou Normal University Hangzhou China; ^2^ Department of Surgery Stanford University Stanford, CA United States; ^3^ HBI Solutions Inc Palo Alto, CA United States; ^4^ Department of Cardiothoracic Surgery Stanford University Stanford, CA United States; ^5^ Clinical and Translational Research Program Betty Irene Moore Children's Heart Center Lucile Packard Children’s Hospital Stanford, CA United States; ^6^ Department of Oncology The First Hospital of Shijiazhuang Shijiazhuang China; ^7^ Tianjin Key Laboratory of Cardiovascular Remodeling and Target Organ Injury Pingjin Hospital Heart Center Tianjin China; ^8^ China Electric Power Research Institute Beijing China; ^9^ School of Management Zhejiang University Hangzhou China; ^10^ HealthInfoNet Portland, ME United States; ^11^ Health Care Big Data Center School of Public Health Zhejiang University Hangzhou China

**Keywords:** hypertension, risk assessment, electronic health records, multiple chronic conditions, mental disorders, social determinants of health

## Abstract

**Background:**

As a high-prevalence health condition, hypertension is clinically costly, difficult to manage, and often leads to severe and life-threatening diseases such as cardiovascular disease (CVD) and stroke.

**Objective:**

The aim of this study was to develop and validate prospectively a risk prediction model of incident essential hypertension within the following year.

**Methods:**

Data from individual patient electronic health records (EHRs) were extracted from the Maine Health Information Exchange network. Retrospective (N=823,627, calendar year 2013) and prospective (N=680,810, calendar year 2014) cohorts were formed. A machine learning algorithm, XGBoost, was adopted in the process of feature selection and model building. It generated an ensemble of classification trees and assigned a final predictive risk score to each individual.

**Results:**

The 1-year incident hypertension risk model attained areas under the curve (AUCs) of 0.917 and 0.870 in the retrospective and prospective cohorts, respectively. Risk scores were calculated and stratified into five risk categories, with 4526 out of 381,544 patients (1.19%) in the lowest risk category (score 0-0.05) and 21,050 out of 41,329 patients (50.93%) in the highest risk category (score 0.4-1) receiving a diagnosis of incident hypertension in the following 1 year. Type 2 diabetes, lipid disorders, CVDs, mental illness, clinical utilization indicators, and socioeconomic determinants were recognized as driving or associated features of incident essential hypertension. The *very high risk* population mainly comprised elderly (age>50 years) individuals with multiple chronic conditions, especially those receiving medications for mental disorders. Disparities were also found in social determinants, including some community-level factors associated with higher risk and others that were protective against hypertension.

**Conclusions:**

With statewide EHR datasets, our study prospectively validated an accurate 1-year risk prediction model for incident essential hypertension. Our real-time predictive analytic model has been deployed in the state of Maine, providing implications in interventions for hypertension and related diseases and hopefully enhancing hypertension care.

## Introduction

### Background

Hypertension affects more than 85 million American adults and over 1.1 billion people worldwide, and elevated blood pressure is one of the leading modifiable metabolic risk factors for noncommunicable diseases and global mortality [1,2]. For example, in some age groups, the risk of cardiovascular disease (CVD) can double with each 20-10 mmHg increase of blood pressure [3]. Therefore, efficient management of blood pressure is a critical step toward reducing morbidity and mortality from chronic diseases such as coronary heart disease and stroke. Moreover, inadequate management of hypertension, including failure to diagnosis, treat, and control high blood pressure, contributes enormous medical, socioeconomic, and human costs [4]. In the United States, the average annual total cost associated with high blood pressure was US $51.2 from 2012 to 2013, with US $37.2 billion in direct costs from health care services and medications and US $3.9 billion in indirect costs owing to lost productivity from morbidity and mortality [1].

Commonly identified risk factors for progression to hypertension include age, gender, body mass index, obesity, stress, lipoproteins, cholesterol, physical inactivity, smoking, and family history [5]. Evidence from clinical trials suggests that early prevention of hypertension with lifestyle modification or drug treatment among individuals without hypertension but having risk factors or prehypertension may reduce the incidence of hypertension in the short-term and for several years after [6-8]. On the other hand, a potentially desirable and operable alternative to universal implementation of such measures is to stratify individuals into different risk groups by applying prediction tools, then target the highest risk segment of the population for subsequent lifestyle intervention or medical treatment, and eventually prevent their progression from high-risk status to actual hypertension [9].

### Possible Limitations of Existing Hypertension Prediction Models

In the last two decades, more than 15 high-quality hypertension risk prediction models were constructed and compared in terms of study design, model performance, calibration ability, as well as impact on decision making and outcomes of care [10]. These models all achieved acceptable to good discrimination with an area under the curve (AUC) between 0.71 and 0.81. However, as they were formed using traditional statistical methods (eg, Cox regression or logistic regression), most existing hypertension prediction models are limited by either small sample size (ie, using data from only a single medical facility), relatively few (<15) traditional risk factors, or lack of ability to monitor real-time changes in predictors and therefore hypertension risk [11].

On the other hand, the use of electronic health records (EHRs) is becoming increasingly common in hospitals and clinics [12]. By tracking almost all aspects of a patient’s care over time, EHR data contain an individual’s integrated and comprehensive clinical history and can be used to build risk models that can potentially help predict disease progression, revealing the evolution of disease, and thus, offering great promise for accelerating clinical research and predictive analysis on a population [13,14]. As the versatility of EHR-based datasets provides more generalized prediction results with high levels of confidence, the clinical construction of EHR-based risk prediction models has become more effective and is in higher demand [15]. Although several EHR-based prediction models have been successfully built for other diseases (eg, type 2 diabetes, kidney disease, and cancer) [14,16-26], such models are still relatively unexplored for the prediction of hypertension.

### Aim of This Study

Given that the EHR data have many dimensions and are usually sparse and subject to random errors, machine learning approaches are recognized as good choices for constructing EHR-based risk models based on their ability to select impactful predictors automatically from hundreds of features and their robustness to feature correlation and random errors [27-29]. Therefore, to take advantage of both EHR data and machine learning approaches, our study aimed to construct and prospectively validate a new hypertension risk prediction model that is able to utilize an individual’s current 1-year clinical information to capture previously ignored but potentially powerful predictors directly from EHRs, including patients’ current health conditions, chronic disease and medication history, clinical utilization measures, and social determinants. It is hoped that this new approach could ultimately predict the probability of receiving a new hypertension diagnosis in the near term (ie, during the next 1 year) with improved accuracy.

## Methods

### The Health Information Exchange Dataset of Maine

**Figure 1:**
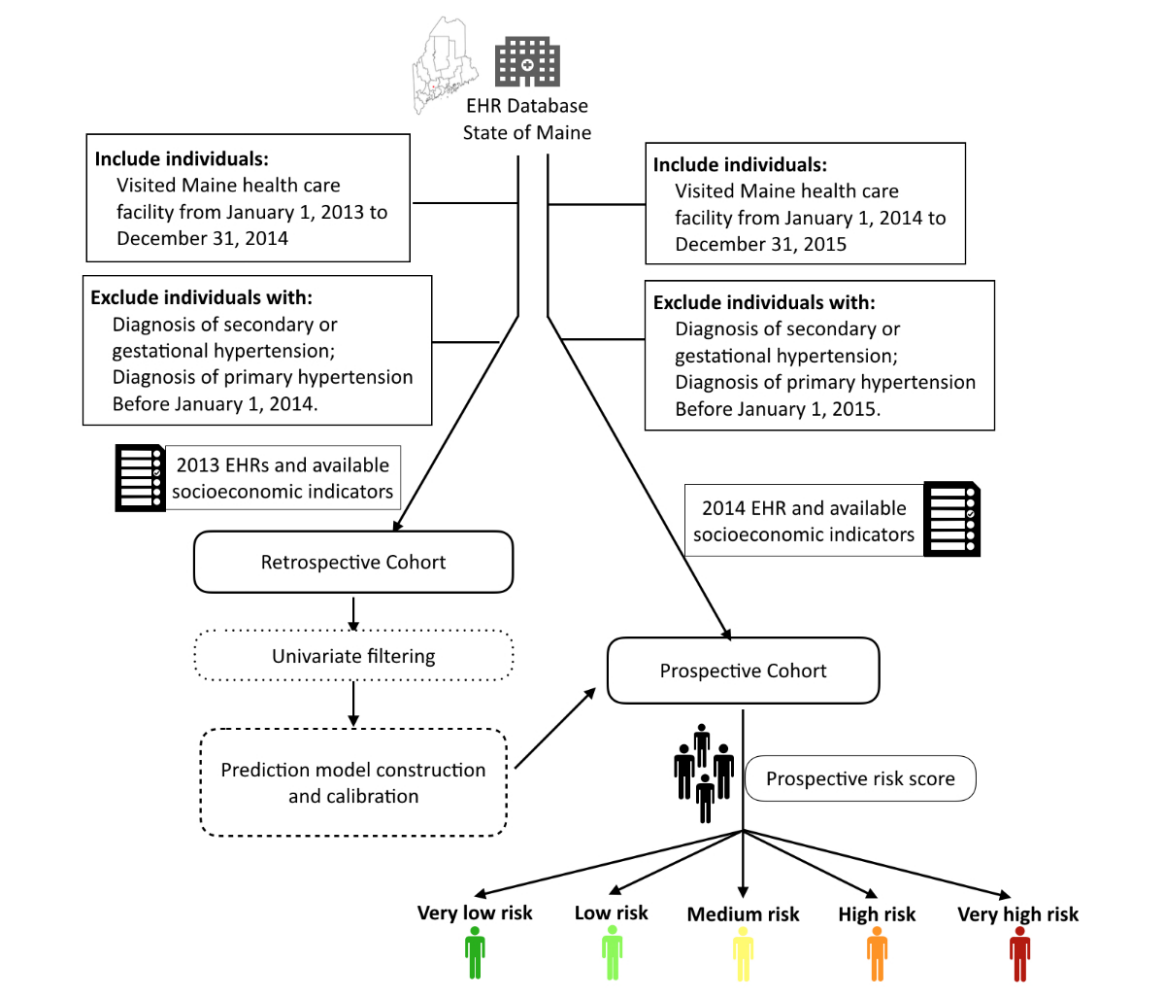
Workflow diagram depicting model construction and evaluation. The retrospective cohort consisted of 823,627 individuals with electronic health record (EHR) profiles extracted from 2013, 92,512 of whom (cases) developed hypertension in 2014. The validation cohort consisted of 680,810 individuals, with EHR profiles extracted from 2014, 60,065 of whom received a new hypertension diagnosis in 2015.

The analyzed dataset was derived from EHRs of all patients that visited any of 35 hospitals, 34 federally qualified health centers, and more than 400 ambulatory practices in the state of Maine from January 1, 2013 to December 31, 2015. This dataset covered almost 95% of the population of Maine and is a subset of the health information exchange (HIE) network provided by the HealthInfoNet organization. This study was approved by the institutional review board of Stanford University, and all personal privacy information was well protected and removed during the process of analysis and publication. Samples were excluded if they had any diagnosis of secondary or gestational hypertension, leaving a total of 1.5 million individuals in the dataset for the analysis. The detailed inclusion and exclusion criteria were demonstrated in the study design workflow (Figure 1).

#### Definition of Hypertension

Hypertension in this study was defined by the International Classification of Diseases, 9th Revision, Clinical Modification (ICD-9-CM) diagnosis codes from category 401, which refers specifically to essential (or primary) hypertension. For the prediction modeling cohort, cases in the retrospective population referred to patients who received a new diagnosis of essential hypertension during the calendar year of 2014 (from January 1, 2014 to December 31, 2014), whereas cases in the prospective cohort were patients receiving an incident diagnosis of essential hypertension during the 2015 calendar year (from January 1, 2015 to December 31, 2015). Along with the implementation of ICD-10-CM since October 1, 2015, an ICD-10-CM category of I10 was used to define the diagnosis of primary hypertension, which is equivalent to the ICD-9-CM category 401 according to the ICD-10-CM General Equivalence Mapping (GEM), a crosswalk between the two code standards maintained by the Center for Medicare Services and the Centers for Disease Control and Prevention [30].

#### Cohorts

Given that our prediction model was constructed to predict an individual’s hypertension risk during the following 1 year based on his or her medical records from the current year, feature profiles for the retrospective cohort were extracted from the clinical and health historical record of 2013, giving a total of 823,627 patients, 92,512 of whom developed hypertension in the year 2014.

Samples were excluded from the retrospective cohort if there was any record of a hypertension diagnosis before January 1, 2014. Similarly, the prospective cohort was extracted from the clinical and health historical record of 2014, offering a total of 680,810 samples and 60,065 cases with incident hypertension in the year 2015. Samples were excluded in prospective cohort if there was any record of a hypertension diagnosis before January 1, 2015.

### Prediction Model Construction and Evaluation

#### Features Preprocessing and Selection

Various categories of data were extracted from the original health records, including demographics, laboratory and radiographic test results, essential and secondary diagnoses and procedures, outpatient medication prescriptions, clinical utility records, as well as a number of accessible socioeconomic variables extracted from the US census and United States Department of Agriculture (USDA) websites [31,32]. Overall, more than 15,000 features were recruited in our original data pool. Among them, laboratory and radiographic test results were coded by logical observation identifier names and codes, outpatient medication prescriptions were coded by the National Drug Code, and primary and secondary diagnoses and procedures were coded by ICD-9-CM. All ICD-10-CM codes were mapped to ICD-9-CM using the GEM tool to keep the data consistent. Some socioeconomic features are directly downloaded from the US census website using the advanced search tool under the *American Fact Finder* category, and others were derived from the USDA website using the Web-based mapping tool, *Food Environment Atlas* (see Multimedia Appendix 1). Using zone improvement plan (ZIP) code or county or tract, all these socioeconomic variables are integrated into our knowledge base.

Before feature selection, the k-nearest neighbors (KNN) approach was used to impute missing data. For a given patient with missing values, the KNN method identified the k-nearest patients based on Euclidean distance. Using these patients, missing values were then replaced using a majority vote for discrete variables and weighted means for continuous features. One advantage of using this method is that missing values in all features are imputed simultaneously without the need to treat features individually [33].

Before the machine-learning algorithm, a univariate correlation filtering step was introduced to remove all features that are not directly related to the outcome variable [34]. In general, the Cochran-Mantel-Haenszel test is applied for binary features by investigating the association between the feature and outcome under age-group strata using the following formula: *R=∑*^K^_i=1_*A*_i_*D*_i_*/T*_i_*/ ∑*^K^_i=1_*B*_i_*C*_i_*/T*_i_, where *A*_i_, *B*_i_, *C*_i_, and *D*_i_ are the counts of individuals in 2x2 contingency table of the *i-* th age group. In addition, the Cochran-Armitage trend test is used to test ordinal variables, and univariate logistic regression is used for continuous variables. After applying the criteria of *P* ≤.05, 798 out of 15,280 features survived from this screening procedure and were recruited in the following stages of XGBoost algorithm.

#### Model Construction

The retrospective cohort was utilized for construction of the prediction model. This process was accomplished in two phases: (1) a data training algorithm to generate predictive estimates and (2) a calibration stage to produce risk scores for each patient.

For the first stage of data training, a supervised machine learning and data mining tool, XGBoost [35], was applied to develop the prediction model based on features survived in the univariate phase. Generally speaking, this algorithm generates an ensemble of classification trees and sums the scores in the corresponding leaves of each tree to calculate a final predictive estimate *ŷ*_i_ for the *i-* th instance (*i=1,..., n*), as demonstrated in equation 1, where each *ƒ*_k_ corresponds to an independent classification tree:

*ŷ*_i_*= ø(x*_i_*) = ∑*^K^_k=1_*ƒ*_k_*(x*_i_*)* (1)

For this study, the depth of each tree was set to be 5 and *K* equaled 500. The model at the *t-* th iteration was trained to minimize the following objective, where *l* is a differentiable convex loss function that not only measures the difference between the target y_i_ and the prediction *ŷ*_i_^(t-1)^ of the *i*-th instance at the *t-1-* th iteration but also takes the *ƒ*_t_ that improves the model most into account:

*L*^(t)^*= ∑*^n^_i=1_*l(* y_i_*, ŷ*_i_^(t-1)^*+ ƒ*_t_*(x*_i_*)) +* Ω (*ƒ*_t_) (2)

The term Ω penalizes the complexity of the regression tree functions to avoid issues of overfitting. The approximate greedy algorithm was adopted to split trees and sort and pick features on each node according to percentiles of distribution. Splitting points were selected to optimize purity at the next splitting level.

For the second phase of calibration, *ŷ* estimates were mapped to positive predictive values (PPVs) in the retrospective cohort. A PPV of a corresponding predictive estimate *ŷ* was defined as the proportion of incident hypertension events in the cohort having predictive estimates the same as or larger than this *ŷ*. Thus, PPVs could also be interpreted as risk scores that measured probability of receiving a new diagnosis of essential hypertension within the next 1 year among individuals having predictive estimates the same as or larger than this *ŷ*.

#### Model Evaluation

In the process of model evaluation, hypertension risk score expressed as PPVs were calculated and assigned to each individual in the prospective cohort. Following that, we further ranked individuals by their risk scores from low to high and allocated them into five distinct risk categories, manifesting individuals’ chance of developing essential hypertension within the next 1 year to be either *very low*, *low*, *medium*, *high*, or *very high*. Performance of the proposed 1-year hypertension risk prediction model was investigated carefully within each risk category in terms of sensitivity, specificity, and PPV. The receiver operating characteristic curve and the validated AUC value were derived to evaluate the performance of the derived prediction model.

For these highly weighted features, age-sex adjusted odds ratios (ORs) between cases and controls were calculated along with 95% CI using logistic regression. Distribution patterns of impactful risk predictors were explored and compared among different risk categories, uncovering predictors’ characteristics under our constructed prediction model. Moreover, survival analyses such as multivariable Cox regression were employed for the purpose of subpopulation comparison. Spearman rank correlations were performed to assess the correlation between socioeconomic features and the next 1-year risk of hypertension.

## Results

### Demographic Baseline

Baseline demographic features and hypertension-related disease conditions in retrospective and prospective cohort, summarized in Table 1, indicate that most characteristics were similarly distributed between the two cohorts. When considering hypertension relevant diagnostic diseases, CVDs, disorders of lipid metabolism, type 2 diabetes, and chronic obstructive pulmonary disease (COPD) were the most common diseases in the nonhypertensive retrospective and prospective cohorts. Furthermore, the distributions of other chronic diseases such as anemia, idiopathic hypersomnia, prehypertension, and chronic kidney disease (CKD) were balanced between the retrospective and prospective populations.

**Table 1:** Baseline characteristics of the retrospective and prospective cohorts.

Characteristic		Training cohort (N=823,627) n (%)	Validation cohort (N=680,810) n (%)
**Age (years)**			
	<35	389,081 (47.23)	403,932 (59.30)
	35-49	202,906 (24.64)	158,580 (23.29)
	50-64	118,624 (14.40)	71,687 (10.53)
	≥65	112,709 (13.68)	46,611 (6.85)
**Gender**			
	Male	366,859 (44.54)	294,430 (43.25)
	Female	456,768 (55.46)	386,380 (56.75)
**Race**			
	White	781,457 (94.88)	634,107 (93.14)
	Black	9060 (1.11)	10,212 (1.50)
	Asian	8978 (1.09)	6332 (0.93)
	Other	24,050 (2.92)	30,159 (4.43)
**Diagnostic disease**			
	Cardiovascular diseases	50,769 (6.16)	26,259 (3.86)
	Disorders of lipid metabolism	22,223 (2.70)	22,063 (3.24)
	Type 2 diabetes	23,464 (2.85)	17,999 (2.64)
	COPD^a^	8391 (1.02)	8534 (1.25)
	Acquired hemolytic anemia	6508 (0.79)	6289 (0.92)
	Liver disorders	6194 (0.75)	4121 (0.61)
	Idiopathic hypersomnia	5141 (0.62)	2991 (0.44)
	Prehypertension	2439 (0.30)	1877 (0.28)
	Chronic nephritis	3055 (0.37)	1713 (0.25)
	Chronic kidney disease	2389 (0.29)	1404 (0.21)
	Hypopotassemia	1245 (0.15)	765 (0.11)
	Hyposmolality or hyponatremia	549 (0.07)	381 (0.06)
	Diagnosed as primary hypertension after 1 year	92,512 (11.23)	60,065 (8.82)

^a^COPD: chronic obstructive pulmonary disease.

### Model Performance

**Figure 2:**
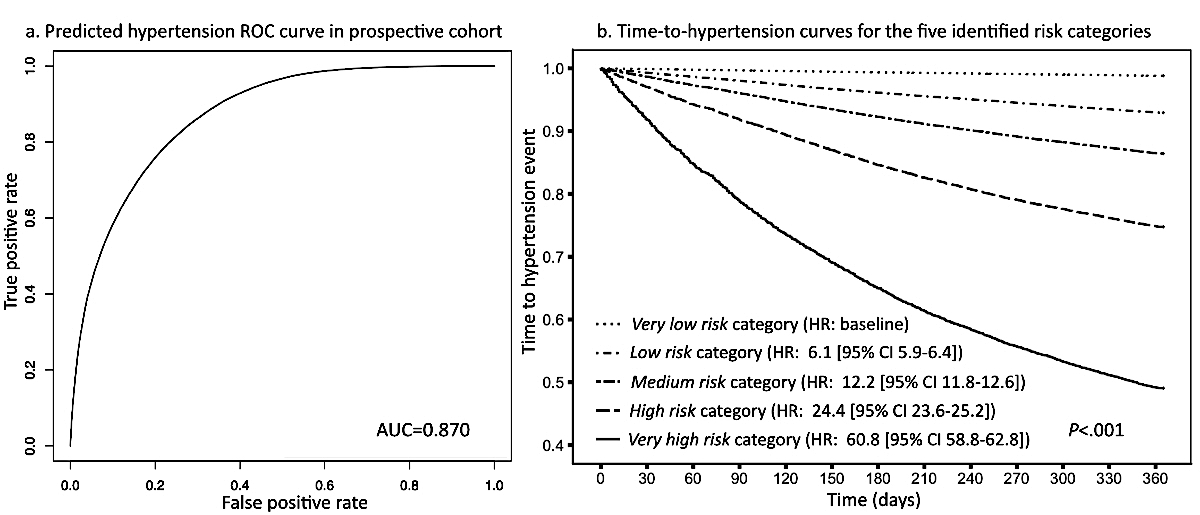
(a) The receiver operating characteristic (ROC) curve derived from the prospective cohort (left). (b) Survival curves (right) of the five risk categories identified by the 1-year hypertension risk prediction model. Five risk categories were defined: very low (risk score<0.05), low (risk score 0.05-0.1), medium (risk score 0.1-0.2), high (risk score 0.2-0.4), and very high (risk score >0.4). HR: hazard ratio.

By adopting the machine learning tool XGBoost on the EHR-based data, our prediction model reached a fitted AUC of 0.917 in the retrospective cohort and a predicted AUC of 0.870 in the independent prospective cohort (Figure 2). A total of 381,544 individuals were labeled as *very low risk* (ie, received risk score 0-0.05), and <1.19% (4526/381,544) were affected by hypertension in the next 1 year. In contrast, for 41,329 individuals identified in the *very high risk* category (ie, risk score >0.4), more than 50.93% (21,050/41,329) received a confirmatory diagnosis of essential hypertension during the next 1 year. Among 60,065 patients with confirmed hypertension in the next 1 year, more than one-third (35.04%, 21,050/60,065) were correctly classified into the *very high risk* category, and only 7.54% (4526/60,065) of them were falsely assigned to the *very low risk* population (see Multimedia Appendix 2).

To further evaluate the model’s discriminative ability based on the five risk categories, we estimated each category’s time-to-hypertension curve using univariable Cox regression. As a result, five distinct survival curves were created and well stratified in terms of hypertension hazard (*P*<.001), giving a hazard ratio (HR) for the *very high risk* category as high as 60.8 (95% CI 58.8-62.8) relative to the *very low risk* group (Figure 2).

**Figure 3:**
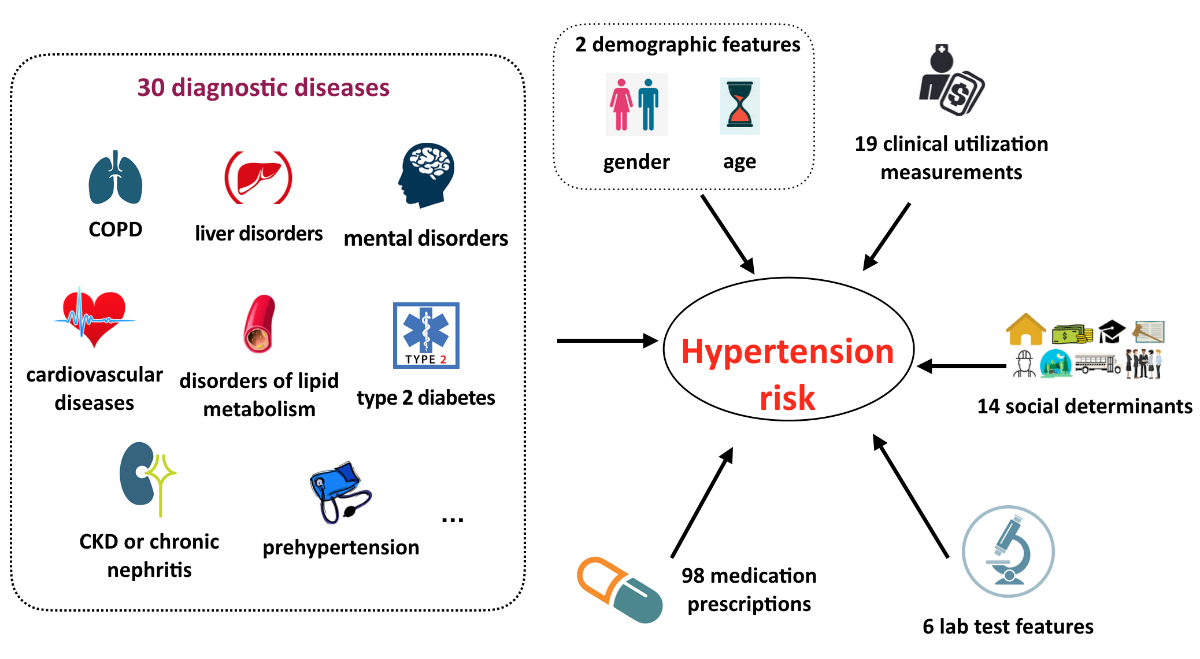
Six classifications of the 169 electronic health record (EHR)–based impactful features recognized by our risk model. COPD: chronic obstructive pulmonary disease; CKD: chronic kidney disease.

With the original pool of 15,280 features merging from EHRs and socioeconomic indicators, 798 features passed the univariate selection phase and were recruited as candidates of predictors for the following machine learning algorithm. During the process of model construction, XGBoost adopted the approximate greedy algorithm to split trees by sorting and picking features on each node to optimize purity at each splitting level. Finally, it automatically recruited a total of 169 features as predictors into the prediction model, consisting of two demographic features, 14 socioeconomic characteristics, 30 diagnostic diseases, six laboratory tests, 98 medication prescriptions, and 19 clinical utilization measures (Figure 3). The most impactful 80 features are listed in Multimedia Appendix 3, as well as their age-sex adjusted ORs or coefficients and 95% CIs derived from the prospective cohort. Besides age and sex, the meaningful features mainly comprised hypertension-related disease diagnoses, medications for these related diseases, clinical utilization measurements, and socioeconomic indicators. For diagnosed diseases, prehypertension, type 2 diabetes, CVDs (combination of congestive heart failure, atherosclerotic heart disease, coronary heart disease, and myocardial infarction), and idiopathic hypersomnia were recognized as the conditions most associated with hypertension, all with an OR>3.00 in prospective cohort. In terms of medication prescriptions, other than those for CVDs, type 2 diabetes, and disorders of lipid metabolism, our prediction model also captured consumed medications for treatment of mental health conditions, including drugs for depression, anxiety, and schizophrenia. From the clinical utilization domain, inpatient admissions and outpatient visits during last year and total estimated health care costs for the patient over last year were significant. Furthermore, social determinants such as education level, type of health insurance, and environmental factors related to dietary habits and physical activities were also detected by the model as powerful predictors of incident hypertension within the next 1 year.

### Significant Features

To further validate the impact of recognized risk factors on the risk of essential hypertension, we carefully investigated the *very high risk* population in the prospective cohort and compared it with the *very low risk* category, revealing the unique characteristics of *very high risk* individuals.

#### Demographic Features

Age and gender were recognized as the most impactful demographic features in our hypertension risk prediction model. To follow the regulations of Health Insurance Portability and Accountability Act, all ages over 89 years were combined into one category to protect patients’ health information.

**Figure 4:**
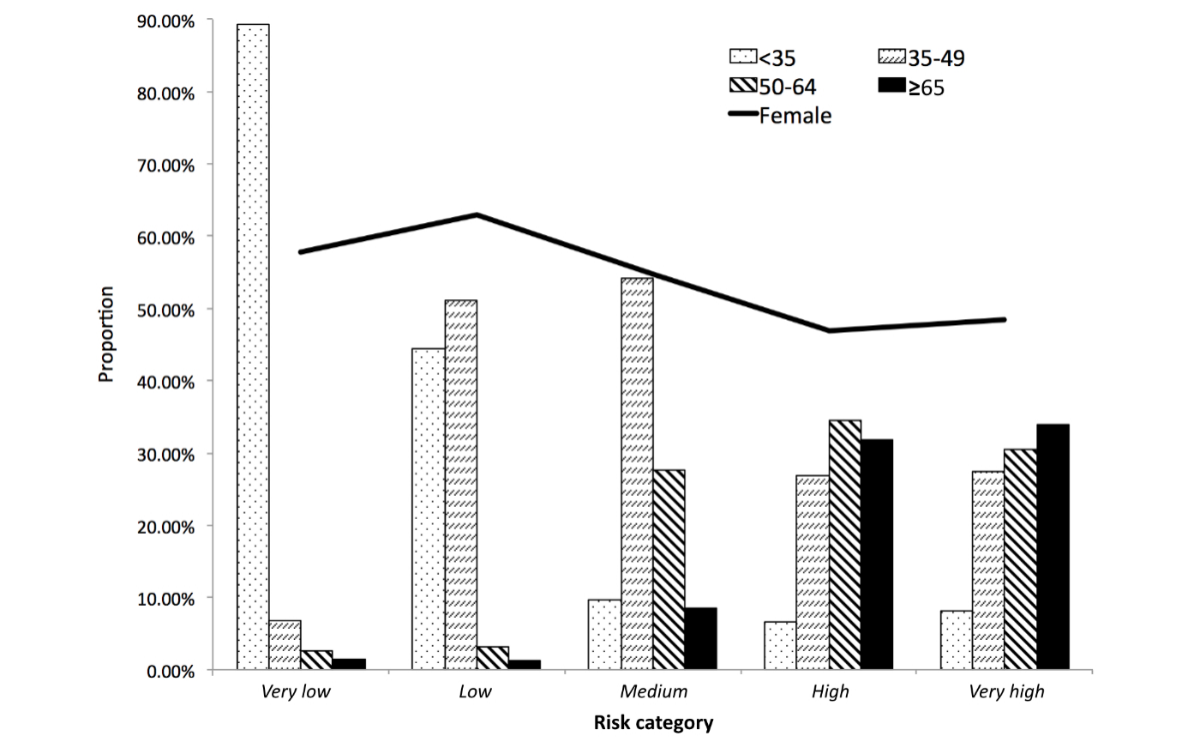
Constituent ratios of age and gender (female) subgroups across the identified five risk categories. Age groups (years): <35, 35-49, 50-64, and ≥65.

To maintain the consistency of this feature, other ages were aggregated into categories as well. In addition, this age categorization could help us capture natural differentials in age-related socioeconomic backgrounds such as work or retirement status and eligibility for pensions and medicare and investigate their impact on hypertension risk and incidence [36]. Therefore, from the *very low risk* to the *very high risk* category of hypertension, constituent ratios were calculated for four distinct age groups and compared with each other (Figure 4). Low-risk categories consisted of young people, whereas high-risk categories were concentrated with older individuals, confirming age group as a strong predictor of hypertension. Younger individuals (<35 years) comprised almost 89.27% (340,621/381,544) of the *very low risk* category. Middle-aged adults (35-49 years) were overwhelmingly dominant in the *low risk* and *medium risk* categories, with proportions of 51.04% (53,372/104,565) and 54.11% (53,792/99,415), respectively. On the contrary, individuals in the age range of 50 to 64 years occupied 34.57% (18,651/53,957) of the *high risk* population, and people aged ≥65 years constituted the largest subset (33.88%, 14,000/41,329) of the *very high risk* population (see Multimedia Appendix 4). Following that, we formed a subpopulation with people ≥65 years and found that our hypertension prediction model reached an acceptable to good discriminative ability with this subpopulation, with an AUC of 0.744 (see Multimedia Appendix 5). On the other hand, females were likely to have lower risk of hypertension than males, comprising majorities (58.25%, 341,072/585,524) of the three relatively low-risk categories (ie, *very low*, *low*, and *medium*) but decreasing to proportions of 46.89% (25,300/53,957) and 48.41% (20,008/41,329) in the *high risk* and *very high risk* groups (Figure 4). In summary, the *very high risk* population mainly comprised aged (>50 years) males, who occupied almost one-third of this category.

#### Diagnosed Chronic Diseases

Most individuals in the *very high risk* population had a history of multiple chronic medical conditions. Most commonly CVDs, type 2 diabetes, and disorders of lipid metabolism affected 25.08% (10,367/41,329), 20.17% (8,336/41,329), and 8.76% (3619/41,329) of the population, respectively (see Multimedia Appendix 4), compared with only 0.65% (2473/381,544), 0.26% (1006/381,544), and 0.27% (1036/381,544) of the *very low risk* cohort.

**Figure 5:**
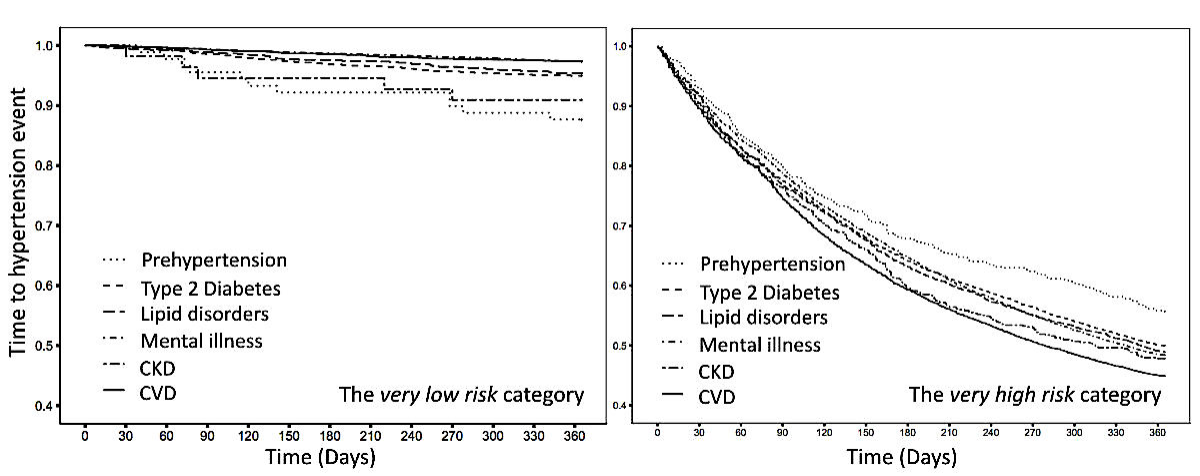
Kaplan-Meier curves depicting time-to-hypertension for the next 1 year according to coexisting disease subgroup for the very low risk (left) and very high risk (right) categories of the prospective cohort. Disease subgroups comprised patients who received diagnoses of either prehypertension, type 2 diabetes, lipid disorders, mental illness, chronic kidney disease (CKD), or cardiovascular disease (CVD), respectively.

To further explore real-time hypertension events in the *very high risk* category, we investigated time-to-hypertension curves in terms of disease subgroups (ie, prehypertension, type 2 diabetes, lipid disorders, mental illness, CKD, and CVDs) and compared them with curves generated from the *very low risk* cohort (Figure 5). In the *very high risk* category, 55.09% (5711/10,367) patients with CVD would receive diagnoses of hypertension in the next 1 year, and this probability stayed around 50% for patients with conditions of either type 2 diabetes, lipid disorders, mental illness, or CKD. Thus, a half of individuals with a chronic disease in the *very high risk* category would develop hypertension within the next 1 year, with CVD patients at the highest risk of hypertension during the next 1 year, whereas prehypertension patients had the lowest risk, implying different causal mechanism of hypertension under different disease scenarios at the *very high risk* stage. In contrast, in the *very low risk* category, more than 85% of patients were free from development of hypertension in the next 1 year, regardless of chronic conditions, where the survival curve dropped fastest for patients with prehypertension but went down slowly for the cardiovascular subgroup, opposite to the trends at the *very high risk* stage.

#### Mental Illness

Apart from medications for CVD, type 2 diabetes, lipid disorders, and COPD, our prediction model also recognized 18 important medications prescribed for mental health diagnoses as powerful predictors of 1-year hypertension risk, which were mainly drugs prescribed for mood disorders such as depression, anxiety disorders, and schizophrenia disorders (see Multimedia Appendix 6). In our original HIE dataset from Maine, patient records related to mental health were initially blocked because of privacy protection issues. Therefore, our study utilized these consumed drugs as proxies to inspect the association between mental illness and risk of hypertension. People under treatment for depression, anxiety, and schizophrenia disorders were significantly enriched in the *very high risk* group for hypertension, with proportions of 22.21% (9181/41,329), 13.40% (5538/41,329), and 2.71% (1119/41,329), respectively, but much less prevalent in the *very low risk* group (see Multimedia Appendix 7).

Taking that most prevalent depression as an example, we further divided the prospective cohort into two subgroups of individuals who did and did not carry other chronic disease diagnoses and validated the association between mental disorders and hypertension risk accordingly.

**Figure 6:**
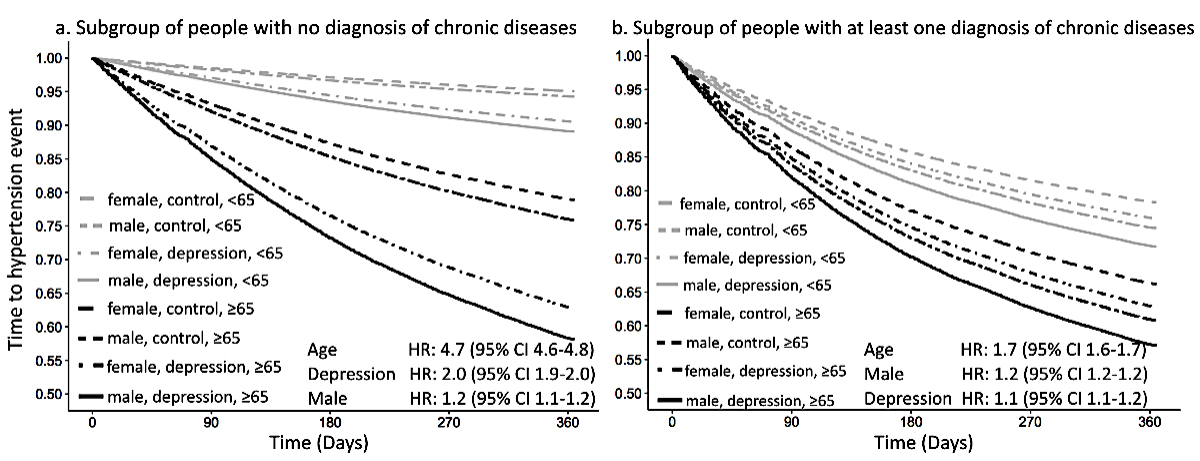
Predicted time-to-hypertension curves for patients (a) who received no diagnosis of other chronic diseases except possible depression and (b) who had at least one diagnosis of other chronic disease. Curves for both subgroups were stratified by age (<65 vs ≥65 years), gender (male vs female), and depression diagnosis (depression vs control).

By using multivariable Cox proportional hazards models, the contribution of depression to the hazard for hypertension was assessed after adjustment for age and gender (Figure 6). In the subgroup that comprised people with other chronic conditions, depression status revealed a relatively small effect on the 1-year hypertension hazard (HR: 1.1 [95% CI 1.1-1.2]), lower than both age and gender. However, in the subgroup of patients without other chronic physical conditions, we found that depression was associated with a two-fold hypertension risk (HR: 2.0 [95% CI 1.9-2.0]). Following that, time-to-hypertension curves were stratified by age (<65 and ≥65 years), depression status, and gender, in order of priority. Age, as the most impactful predictor, attained the highest HR (HR: 2.0 [95% CI 4.6-4.8]), whereas depression status was the second impactful feature. Therefore, as depicted in the two lowest survival curves in Figure 6, for individuals ≥65 years (mostly assembled in the *very high risk* category), a diagnosis of depression would double the 1-year hazard of hypertension even if there were no other chronic conditions, regardless of gender.

#### Clinical Utilization Indicators

More severe health conditions are expected to consume more resources. Accordingly, clinical utilization indicators in our study revealed similar patterns to those highlighted diseases when compared across five risk categories. These utilization parameters such as outpatient visits, inpatient admissions, clinical cost, total number of consumed prescriptions, and total number of laboratory tests in the past year were lowest in the *very low risk* group but gradually increased from low to high risk groups, with the highest values in the *very high risk* population (see Multimedia Appendix 4).

**Figure 7:**
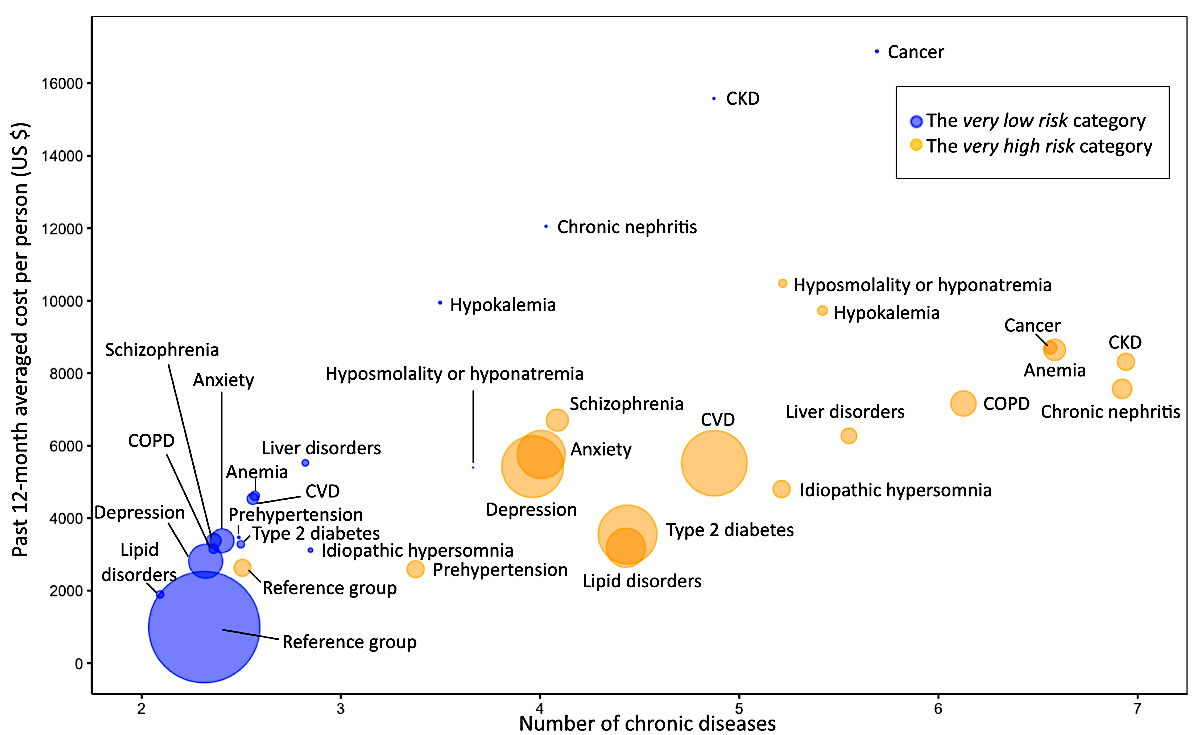
Patients’ average clinical costs in the past 12 months against the average number of chronic diseases. The balls were formed by 16 common disease subgroups under the very low risk (blue balls) and very high risk (yellow balls) categories, respectively. The ball size indicates the proportion of the disease subgroup under this risk category. The 16 chronic diseases were prehypertension, cardiovascular disease (CVD), type 2 diabetes, lipid disorders, chronic kidney disease (CKD), chronic nephritis, depression, anxiety, schizophrenia, chronic obstructive pulmonary disease (COPD), liver disorders, cancer, anemia, hypokalemia, idiopathic hypersomnia, and hyposmolality or hyponatremia. Reference groups consisted of patients with no diagnosis of any above chronic diseases.

On the basis of these findings, we focused compared patients in the *very high risk* and *very low risk* categories with respect to average clinical costs in the prior 12 months for 16 chronic disease subgroups, coordinated by their averaged number of chronic diseases (Figure 7). In the *very low risk* category, patients with prehypertension, CVDs, type 2 diabetes, lipid disorders, and mental disorders had relatively low prevalence (ie, small balls in Figure 7) and clustered in the lower left corner (ie, the low-cost and low-chronic-complexity area), very close to the largest bubble representing the *reference* subgroup formed by nonchronic disease individuals. However, in the *very high* hypertension-risk category, people usually suffered from multiple chronic conditions, resulting in more disease comorbidities and more clinical costs, bubbling toward the upper right corner. Moreover, in the *very high risk* category, the clinically costly CKD and chronic nephritis were recognized as the most severe chronic diseases on average, accompanied by six other chronic conditions, whereas CVD, type 2 diabetes, lipid disorders, and mental disease stayed in a moderate level of disease comorbidity and clinical cost.

#### Social Economic Features

**Figure 8:**
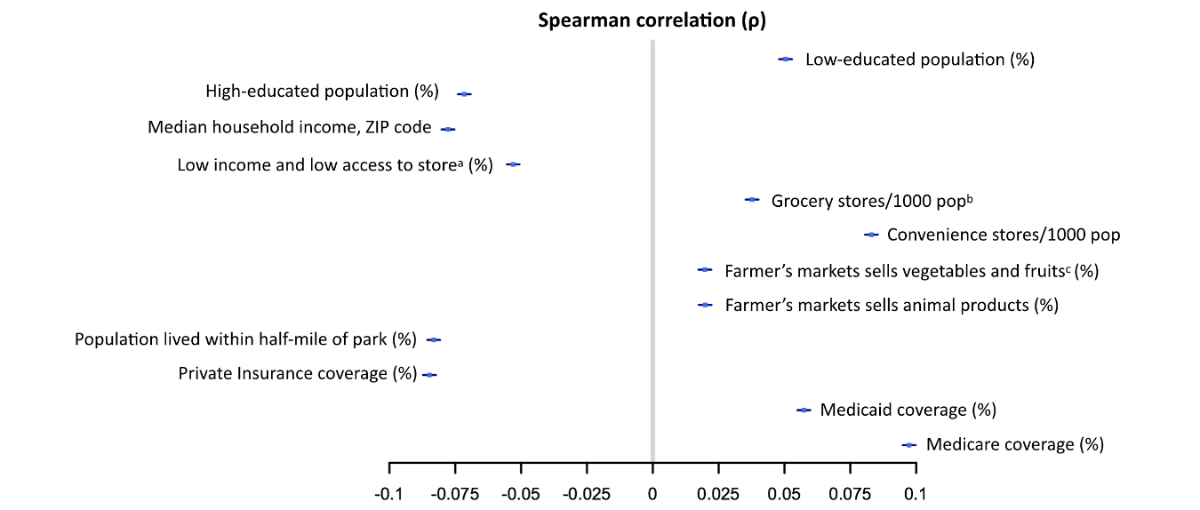
Spearman rank correlation between 12 socioeconomic features and prospective hypertension risk scores. (a) Percentage of people in a county with low income and living more than 1 mile from a supermarket or large grocery store if an urban area, or more than 10 miles from a supermarket or large grocery store if a rural area. (b) The number of supermarkets and grocery stores in the county per 1000 county residents. (c) Percentage of farmers’ markets in the county that sell fresh fruits and vegetables.

Several interesting results were also found in socioeconomic features. In this study, most socioeconomic features were derived from ZIP code or county-based census and USDA data, and thus, they were recognized as community-level social and environmental indicators. The association between significant socioeconomic factors and the summarized hypertension risk scores were further investigated by Spearman rank correlations (Figure 8). Educational disparities revealed a unique and significant correlation with hypertension risk scores in our prediction model. The percentage of the low-education population (ie, a combination of the 18-24 year old population with less than high school graduate diploma and ≥25-year-old population with less than 12th grade diploma in the area) gradually increased from low- to high-risk categories and ultimately displayed a positive correlation (ρ=.05) with hypertension risk, occupying 8.73% (33,310/381,544) and 9.20% (3804/41,329) of the *very low risk* and the *very high risk* subsets (see Multimedia Appendix 8). In contrast, the averaged proportion of high-educated people that received college or associate’s degree or bachelor’s and higher degree grew in an opposite direction and showed a negative correlation (ρ=−.072) with hypertension risk, by attaining its highest percentage of 49.05% (187,148/381,544) in the *very low risk* group and decreased to its lowest value of 47.85% (19,776/41,329) in the *very high risk* population (see Multimedia Appendix 8).

More disparities were found in other social and economic indicators, both community-level persecutors of and defenders against hypertension (Figure 8). Those risk indicators with positive correlations (ρ>0) mainly comprised measures of nearby available food stores (eg, number of grocery and convenience stores per 1000 population and percentages of farmers’ markets selling fruit and vegetables and animal products within a certain region) and the percentage of people benefiting from Medicaid and Medicare type health insurance. On the other hand, the population-based defenders of hypertension (ρ<0) were identified as the median household income, percentage of people with low income and living far from supermarket or large grocery store (>1 mile for an urban area and >10 mile for a rural area), percentage of the population living within a half-mile of NAVTEQ-based parks, and percentage of the population covered by private health insurance, as the higher those values were, the lower the risk of hypertension would be in the population. In summary, in terms of social determinants, the *very high risk* population was likely to be occupied by low-income and low-educated people who benefited from public insurance and lived in an area near food stores but far from parks.

## Discussion

### Summary of Main Findings

In this study, we have prospectively validated a risk prediction model of future 1-year incident essential hypertension using EHR data derived from more than 1.5 million people in the state of Maine. The model achieved 0.917 and 0.870 predictive accuracy in retrospective and prospective (validation) cohorts, respectively. On the basis of the risk model, patients were ultimately stratified into five distinct risk categories, ranking the hypertension risk as *very low*, *low*, *medium*, *high*, or *very high*. In the prospective (validation) cohort, these five identified risk categories showed distinct HRs for incident hypertension within the next 1 year (Figure 2), indicating our model’s ability to target those most at risk for subsequent prevention management.

Unlike traditional prediction methods, our study adopted a machine learning algorithm called XGBoost for feature selection and model construction. This supervised machine learning technique is designed to discover statistical patterns in high-dimensional and multivariate datasets and is able to handle nonlinear correlations and random errors both in input features and the output variable [28].

Variable correlation is another common issue when using a large number of variables spontaneously to construct a model. For instance, in our study, potential spatial correlation may exist among demographic or socioeconomic features. Most regression algorithms such as generalized linear model assume feature independence and could become less accurate if variable correlation or multicollinearity exist. On the contrary, decision trees (including XGBoost) are nonparametric algorithms [37] that do not assume a functional relationship between outcome and features as is required by linear regression models. They naturally perform a greedy algorithm of finding the best splits in the data that maximize the entropy reduction of the outcome during each split. Therefore, once a feature is chosen by XGBoost, the importance of any highly correlated feature will be significantly reduced as the effective split is already achieved by the original feature; accordingly, correlated features will no longer effectively reduce the entropy of the outcome. As a result, XGBoost and other decision tree algorithms are robust to correlated features.

As a result, XGBoost provided a more accurate prediction model (AUC of 0.87 in the prospective cohort) than prior models with no extra cost by capturing previously ignored but potentially powerful predictors from patients’ current health conditions, chronic disease and medication history, clinical utilization measures, and social determinants. Specifically, individuals with the highest risk in our study were typically older (>50 years) and suffered from multiple chronic conditions (concurrence of type 2 diabetes, lipid disorders, mental illness, cardiovascular disease, etc). In the socioeconomic domain, low-income and low-educated people who had public insurance and lived near food stores but far away from parks were enriched in the *very high risk* category. In the prospective cohort, 50.93% (21,050/41,329) of people in the *very high risk* group were diagnosed with hypertension during the next 1 year. Such a high incidence of hypertension in the *very high risk* subgroup made us believe that a short-term (1 year) prediction of hypertension was necessary to identify a high-risk cohort, as well as promote their follow-up intervention or prevention.

### Interpretation of Meaningful Risk Predictors and its Implications for Prevention and Early Intervention

#### Multiple Chronic Conditions

Most individuals in the *very high risk* group suffered from other medical conditions, and only 1.56% (645/41,329) had no *other* chronic medical conditions, whereas the majority (73.10%, 278,923/381,544) of the *very low risk* population had no *other* chronic disease diagnosis. Moreover, as the number of people with diagnostic chronic diseases increased, those with multiple chronic conditions (MCCs), also known as concurrent chronic conditions [38], became very common in the *very high* risk category (Figure 7), occupying almost a third (29.60%, 12,234/41,329) of the population, which also led to a dramatically increased burden of health care utilization and cost (Figure 5). In recent years, epidemiological studies indicated the high-level and continually increased prevalence of MCC worldwide. About a third of the US population suffer from MCC, the majority of which are working-age adults (45-64 years), whereas the prevalence is almost 80% among people ≥65 years [39,40]. Consistent with these findings, our *very high* hypertension risk category could be verified as an MCC population mainly consisting of elderly people with comorbidity of multiple chronic diseases. Hypertension is usually recognized as a major risk factor for cardiovascular and renal diseases. Conversely, within a complex reciprocal interrelationship, both essential and secondary hypertension can also be a consequence of chronic conditions [41,42], especially for elderly people [43]. Therefore, based on our prediction model, high risk of hypertension can be seen as the consequence of a long-term interplay of multiple chronic conditions via possible mechanisms such as polyunsaturated omega-3 free fatty acid deficiency, vascular endothelial dysfunction, immune dysregulation, and unhealthy lifestyle [44,45].

In addition, the definition of prehypertension varied in different studies from time to time [46-48]. In the sixth report of the Joint National Committee (JNC), prehypertension was defined as a systolic pressure of 130 to 139 mm Hg or a diastolic pressure of 85 to 89 mm Hg, whereas the updated seventh report of the JNC (JNC 7) defines prehypertension as a systolic pressure of 120 to 139 mm Hg or a diastolic pressure of 80 to 89 mm Hg. In our study, we derived the feature prehypertension from the ICD-9-CM category of 796.2 and the ICD-10-CM category of R03.0, defined according to JNC 7. Therefore, caution is needed when comparing our findings with those of previous studies, considering the different definitions of prehypertension.

#### Mental Illness

The strength of association between mental disorders and subsequent onset of hypertension has been of interest for a long time. Much work has been done on specific psychological domains of depression, anxiety, impulsive eating disorders, and other mental disorders [49,50]. Furthermore, several studies have focused on the effects of mental disorders on subsequent hypertension [51,52]. Recognizing the impact of mental disorders on the onset of hypertension is clinically meaningful for several reasons. First, both the strength of effect and potential mechanisms of psychological and mental disorders to cause physical health conditions remain unclear [53,54]. Second, such association studies are driven by the ongoing worldwide increase of morbidity related to mental disorders.

In our study, the 1-year hypertension risk model and the depression-based survival analysis provided strong evidence of such an association, especially for individuals having no other chronic physical conditions. That is, about 32.17% (1226/3811) of elderly (≥65 years) patients having no chronic conditions but undergoing treatment for depression would develop hypertension within the next 1 year. EHR data in the state of Maine coding mental health diagnoses were masked for privacy protection; only consumed mental disorder drugs could be accessed as proxies. Therefore, as a limitation of the study, we cannot directly ascertain the effect of mental health disorders or the treatment thereof on the progression of hypertension. However, we can examine the relationship between mental illness diagnoses or therapeutics and hypertension development in several different ways according to the large amount of previous studies exploring this relationship [49-52,55-59]. First, mental illness itself could be a risk factor of hypertension, where possible mechanisms could relate to internal responses from altered sleep patterns, sympathoadrenal hyperreactivity, various neurotransmitter abnormalities, or altered inflammatory processes [55,56], as well as exposure to similar risk factors (eg, early childhood adversity) [57]. It is reported that depression can trigger deregulation in the sympathetic nervous system and hypothalamic-pituitary-adrenal axis, causing severe consequences such as metabolic syndrome and elevated risk of hypertension [59]. Second, mental illness medication could also independently increase the risk of hypertension through various drug-related side effects, which are connected to increasing rates of unhealthy lifestyles such as smoking, alcohol intake, physical inactivity, and drug addiction [45]. Certain drug treatments could also lead to the reprogramming of hypertension-related metabolic pathways. Previous cohort studies have found an increase in hypertension risk when tricyclic antidepressants are used; a possible mechanism is the effect of the aforementioned antidepressants on vagal control over the heart [58]. Future work is needed to address the causal relationship between mental illness and hypertension.

#### Social Determinants

Social determinants of health are defined as “the structural determinants and conditions in which people are born, grow, live, work, and age” [60,61] and include social or environmental supportive factors of health, such as socioeconomic status, education level, employment and income, the physical environment, and access to health care. It has been reported that health disparities and inequalities in these social factors, especially education and poverty, accounted for over a third of total deaths in the United States [62]. Social determinants of health could be collected from global, national, and community levels, manifesting varying localized policies and actions on health promotion. Consistent with previous studies [63-66], our model demonstrated that the hazard of 1-year incident hypertension increased as the median household income and the percentage of high-educated population within a ZIP code area went down. In fact, lower education level could be translated as one of the initial driving and predisposing social features, leading to disparities of subsequent incident hypertension. That is, low education level could directly affect family income and ultimately increase the likelihood of having public health insurance, such as Medicaid or Medicare. In addition to those common social determinants of health, some lifestyle-related social factors were also identified in our study, namely, indicators of nearby accessible food stores and parks. The location of food stores and the types of products sold could affect underlying unhealthy diet habits, whereas environmental supportive facilities such as the concentration of parks or playgrounds in the living area may directly shape people’s physical activities and ultimately decrease hypertension risk. Another interesting finding was that, although low income level would increase hypertension risk overall, lower income populations had reduced hypertension risk if they lived far from food or grocery stores, implying an independent and critical impact of food consumption habits on hypertension regardless of income level. As the majority of the Maine population is white, our study failed to find any significant racial and ethnic disparities for hypertension risk, and it is possible that that our model is not as accurate with regard to individuals of other race or ethnicity. Another limitation of our study is that such community-level social determinants may potentially be less precise and could leave some useful social determinants uncaptured in the risk model.

### Application of the Risk Model

Beyond the prediction of hypertension risk itself, subsequent actions to prevent and intervene are necessary in high-risk patients. In our *very high risk* category, 21.26% (8788/41,329) of the population would receive a diagnosis of hypertension within the first 3 months of next year, and this number would gradually increase to 50.93% (21,050/41,329) after the 1-year period. Therefore, monitoring those high-risk patients as soon as possible and developing a personalized longitudinal intervention plan is important to prevent or delay the development of incident hypertension, as well as to reduce corresponding health care expenditures. Given the fact that 98.44% (40,684/41,329) people in the *very high risk* cohort had other chronic diseases, one possible early intervention plan could be more active monitoring and treatment of relevant diseases, blocking possible pathways from chronic diseases to hypertension development. For instance, strong evidence has illustrated that patients with depressive disorders can be effectively treated in primary care by using either low-cost remote cognitive behavioral therapy or face-to-face psychological interventions, which may ultimately benefit the prevention of hypertension [67]. Moreover, it is also illustrated in our study that a high concentration of parks or playgrounds in the living area can reduce hypertension risk, most likely by shaping people physical activities, implying the importance of community level intervention such as increased supportive neighborhood environments to advocate more healthy lifestyles.

### Study Limitations

Our study has several limitations. First, missing data is inevitable in EHR records, and thus, data organization and extraction are critical. The KNN approach was used in our data preprocessing for missing data imputation. One limitation of this approach is that, when most variables are missing for a certain patient, the KNN cannot be identified appropriately, and therefore, the imputation can be less accurate and cause bias [68]. Second, by directly using EHR data, it is possible that some patients with hypertension failed to have a record of this diagnosis, leading to an underestimation of hypertension prevalence in the study. Third, as the primary and secondary diagnoses and procedures were coded in ICD-10-CM after October 1, 2015, we mapped them back to ICD-9-CM using the GEM tool to keep the data consistent. This mapping strategy may be inadequate as ICD-10 has more than 65,000 codes, whereas ICD-9-CM has only 13,000. Immediately after the ICD-10-CM transition on October 1, 2015, the large number of codes and the increased specificity could have been potential challenges for physicians in terms of code assignment, leading to potential systematic bias [69]. Fourth, some traditional risk factors could not be directly captured by structured EHR data. Future natural language processing [70] could utilize the unstructured EHR data to extract additional risk factors including individual-level lifestyle information (eg, diet habit and physical activity).

### Conclusions

In summary, we have constructed and prospectively validated a risk prediction model of future 1-year incident essential hypertension using EHR data derived from more than 1.5 million people in the state of Maine. The EHR-based model achieved 0.917 and 0.870 predictive accuracy in retrospective and prospective (validation) cohorts, respectively, and successfully stratified patients into five distinct risk categories from *very low*, *low*, *medium*, *high*, to *very high*. Our real-time predictive analytic model has already been deployed in the state of Maine. Integration of such predictive analysis into clinical prescriptive solutions may help health care providers target high-risk populations, tailor the prescription and intensity of treatment solutions to such high-risk cohorts, improve decision making and patient adherence to prescribed intervention, and eventually benefit individuals’ health and quality of life while reducing health care costs.

## Appendix

Multimedia Appendix 1 List of social determinant variables downloaded from the US census and United States Department of Agriculture (USDA) websites, detailed in the data source and mapping method.

Multimedia Appendix 2 The performance of the 1-year hypertension risk prediction model in the prospective cohort, summarized in PPV, sensitivity, and specificity.

Multimedia Appendix 3 The most impactful 80 features selected by our hypertension prediction model.

Multimedia Appendix 4 Distribution of impactful risk predictors across the five risk categories, specified as features of demographics, diagnosed diseases, and clinical utilization.

Multimedia Appendix 5 ROC curves and AUC values of subgroups.

Multimedia Appendix 6 Distribution of impactful medications of depression, anxiety, and schizophrenia across the five risk categories.

Multimedia Appendix 7 Constituent ratios of three mental diseases (depression, anxiety, and schizophrenia) in the very low risk and very high risk categories.

Multimedia Appendix 8 Distribution of social determinant indicators across the five categories with very low, low, medium, high, and very high risk of hypertension.

## Abbreviations

AUC: area under the curve
COPD: chronic obstructive pulmonary disease
CKD: chronic kidney disease
CVD: cardiovascular disease
EHR: electronic health record
GEM: General Equivalence Mapping
HIE: health information exchange
HR: hazard ratio
ICD-9-CM: International Classification of Diseases, 9th Revision, Clinical Modification
JNC: Joint National Committee
KNN: k-nearest neighbors
MCC: multiple chronic conditions
OR: odds ratio
PPV: positive predictive value
USDA: United States Department of Agriculture
ZIP: zone improvement plan
