# Supplementary material for: Prediction of Incident Hypertension Within the Next Year: Prospective Study Using Statewide Electronic Health Records and Machine Learning
Source: J Med Internet Res. 2018 Jan 30;20(1):e22. doi: 10.2196/jmir.9268 (PMC5811646; doi:10.2196/jmir.9268)
Supplement: Multimedia Appendix 1 [file jmir_v20i1e22_app1.pdf]

Appendix 1. List of social determinant variables downloaded from the US census and United States Department of Agriculture (USDA) websites, detailed in the data source and mapping method

Num.	Social Determinants	SD Levels	Variable Type	Mapping Method	Data Source
1	Racial Demographics	Four levels: White; Black/African American; American Indian; Asian	%	ZIP code	US Census ACS (American Community Survey)
2	Urban or Rural	Two levels: Urban ratio; Rural ratio	%	ZIP code	
3	Nativity and Citizenship		%	ZIP code	
4	Education level	Four levels: Less than High school; High School; College associate bachelor; Graduate degree.	%	ZIP code	
5	English proficiency		%	ZIP code	
6	Health insurance status [6]	Six levels: Public: Medicare; Medicaid; VA health care. Private: Employ-based; Direct-purchase; Tricare/military.	%	ZIP code	
7	Poverty or Household income		US $	ZIP code	
8	Unemployment rate		%	ZIP code	

9	Respiratory Hazard Index		[0,+]	County level	US Environmental Protection Agency (EPA)
10	Percentage of population living within half mile to park		%	County level	Centers for Disease Control and Prevention, Community Health Status Indicators (CDC CHSI)
11	GINI inequality index		[0,1]	ZIP code	US Census (ACS)
12	Social Vulnerability Index		[0,1]	County level	CDC or Agency for Toxic Substances and Disease Registry (ATSDR)
13	Low income & low access to store		%	County level	USDA (Food Environment Atlas)
14	Grocery stores/1000 pop		[0,+]	County level	
15	Convenience stores/1000 pop		[0,+]	County level	
16	Farmers' markets selling fruit and vegetables		%	County level	

17	Farmers' markets selling animal products		%	County level	
18	Seniors, low access to store		%	County level	
19	SNAP-authorized stores/1000 pop		[0,+]	County level	
20	Fast-food restaurants/1000 pop		[0,+]	County level	
21	Full-service restaurants/1000 pop		[0,+]	County level	
22	SNAP benefits per capita		US $/capita	County level	
23	Direct farm sales per capita		$	County level	
24	Farmers' markets/1000 pop		[0,+]	County level	
25	Farmers' markets that report accepting SNAP		%	County level	
26	Farmers' markets that report accepting SFMNP		%	County level	
27	Adult diabetes rate		%	County level	
28	Adult obesity rate		%	County level	
29	Recreation and fitness facilities/1000 pop		[0,+]	County level	
30	Recreation and fitness facilities/1000 pop		% change	County level	
31	ERS natural amenity index		0 or 1	County level	

32	Poverty rate		%	County level	
