# Supplementary material for: Prediction of Incident Hypertension Within the Next Year: Prospective Study Using Statewide Electronic Health Records and Machine Learning
Source: J Med Internet Res. 2018 Jan 30;20(1):e22. doi: 10.2196/jmir.9268 (PMC5811646; doi:10.2196/jmir.9268)
Supplement: Multimedia Appendix 2 [file jmir_v20i1e22_app2.pdf]

Appendix 2. The performance of the 1-year hypertension risk prediction model in the prospective cohort, summarized in PPV, sensitivity, and specificity

Risk category	Very low	Low	Medium	High	Very high	Total
Intervals	[0, 0.05]	[0.05, 0.1]	[0.1, 0.2]	[0.2, 0.4]	[0.4, 1]	
Total, n	381,544	104,565	99,415	53,957	41,329	680,810
Case, n	4,526	7,373	13,492	16,491	21,050	60,065
PPV, %	1.19	7.05	13.57	25.25	50.93	
Sensitivity, %	7.54	12.28	22.46	22.68	35.04	
Specificity, %	60.74	15.66	13.84	6.04	3.27	
