# Supplementary material for: Prediction of Incident Hypertension Within the Next Year: Prospective Study Using Statewide Electronic Health Records and Machine Learning
Source: J Med Internet Res. 2018 Jan 30;20(1):e22. doi: 10.2196/jmir.9268 (PMC5811646; doi:10.2196/jmir.9268)
Supplement: Multimedia Appendix 3 [file jmir_v20i1e22_app3.pdf]

Appendix 3. The most impactful 80 features selected by our hypertension prediction model

Characteristic	Prospective cohort (N=680,810)	Control (N=620,745) n (%)	Case (N=60,065) n (%)	OR (95% CI)
**Age**				
<35	403,932	392,550 (63.24%)	11,382 (18.95%)	0.136 (0.133-0.139)
35-50	158,580	138,159 (22.26%)	20,421 (34.00%)	1.799 (1.767-1.832)
50-65	71,687	56,432 (9.09%)	15,255 (25.40%)	3.404 (3.336-3.474)
≥65	46,611	33,604 (5.41%)	13,007 (21.65%)	4.829 (4.722-4.939)
**Gender**				
Female	386,380	354,791 (57.16%)	31,589 (52.59%)	0.832 (0.818-0.846)
**Diseases**				
Cardiovascular diseases	26,259	17,789 (2.87%)	8,470 (14.10%)	2.851 (2.767-2.938)
Disorders of lipid metabolism	22,063	17,353 (2.80%)	4,710 (7.84%)	1.730 (1.669-1.792)
Type 2 diabetes	17,999	11,898 (1.92%)	6,101 (10.16%)	3.935 (3.801-4.073)
COPD[a]	8,534	6,808 (1.10%)	1,726 (2.87%)	1.756 (1.658-1.860)
Liver disorders	4,121	3,397 (0.55%)	724 (1.21%)	1.905 (1.749-2.073)
Idiopathic hypersomnia	2,991	2,295 (0.37%)	696 (1.16%)	2.998 (2.737-3.280)
Prehypertension	1,877	1,281 (0.21%)	596 (0.99%)	4.528 (4.072-5.029)
Chronic nephritis	1,713	1,059 (0.17%)	654 (1.09%)	2.417 (2.174-2.685)
Chronic kidney disease	1,404	876 (0.14%)	528 (0.88%)	2.375 (2.113-2.668)
Hypopotassemia	765	576 (0.09%)	189 (0.31%)	2.482 (2.070-2.965)
Hyposmolality or hyponatremia	381	267 (0.04%)	114 (0.19%)	2.015 (1.586-2.546)
**Medications of Depression**				
Sertraline hcl	19,347	16,841 (2.71%)	2,506 (4.17%)	1.591 (1.519-1.665)
Bupropion hcl	14,166	12,156 (1.96%)	2,010 (3.35%)	2.100 (1.997-2.208)
Trazodone hcl	12,610	10,571 (1.70%)	2,039 (3.39%)	2.053 (1.950-2.160)
Citalopram hydrobromide	10,454	8,964 (1.44%)	1,490 (2.48%)	1.663 (1.567-1.764)
Escitalopram oxalate	8,990	7,794 (1.26%)	1,196 (1.99%)	1.760 (1.648-1.879)

Amitriptyline hcl	7,424	6,185 (1.00%)	1,239 (2.06%)	2.262 (2.117-2.414)
Paroxetine hcl	5,155	4,253 (0.69%)	902 (1.50%)	1.958 (1.811-2.115)
Mirtazapine	3,972	3,290 (0.53%)	682 (1.14%)	1.771 (1.617-1.936)
Nortriptyline hcl	1,849	1,537 (0.25%)	312 (0.52%)	2.115 (1.855-2.405)
**Medications of Anxiety Disorders**				
Lorazepam	13,027	10,906 (1.76%)	2,121 (3.53%)	1.792 (1.703-1.885)
Escitalopram oxalate	8,990	7,794 (1.26%)	1,196 (1.99%)	1.760 (1.648-1.879)
Diazepam	8,838	7,503 (1.21%)	1,335 (2.22%)	1.990 (1.869-2.117)
Alprazolam	8,205	6,725 (1.08%)	1,480 (2.46%)	2.094 (1.970-2.225)
Buspirone hcl	4,192	3,614 (0.58%)	578 (0.96%)	2.271 (2.068-2.489)
**Medications of schizophrenia**				
Aripiprazole	4,671	4,153 (0.67%)	518 (0.86%)	2.075 (1.886-2.278)
Quetiapine fumarate	3,927	3,409 (0.55%)	518 (0.86%)	1.746 (1.580-1.925)
Olanzapine	1,943	1,677 (0.27%)	266 (0.44%)	1.614 (1.404-1.850)
Ziprasidone hcl	863	733 (0.12%)	130 (0.22%)	2.782 (2.286-3.358)
**Utilization**				
Inpatient admissions last year	39,918	34,161	5,757	1.250 (1.211-1.290)
Outpatient visits last year	607,235	550,066	57,169	2.014 (1.937-2.095)
Patient's estimated cost last year (US $)	1,893.12	1,794.14	2,916.03	677.010(571.718-782.302)
Inpatient length of days last year	5.49	5.48	5.55	-0.039 (-0.092-0.013)
Number of chronic disease	2.52	2.46	3.07	0.337 (0.324-0.350)
Number of different medications	7.14	6.91	9.52	2.339 (2.286-2.392)
Total number of medications	22.01	21.10	31.47	9.695 (9.446-9.943)
Total number of abnormal lab test	0.21	0.20	0.34	0.109 (0.105-0.112)
**Social Determinants**				
Low-educated population (%)	8.82	8.79	9.13	0.596 (0.562-0.629)
High-educated population (%)	48.74	48.81	48.02	-0.965 (-1.029--0.900)
Low income and low access to store[a] (%)	3.80	3.81	3.72	-0.024 (-0.034--0.013)
Grocery stores/1000 pop[b]	0.27	0.27	0.27	0.004 (0.004-0.005)
Convenience stores/1000 pop	0.68	0.68	0.70	0.021 (0.020-0.023)

Farmers' markets selling fruit and vegetables[c] (%)	61.30	61.09	63.54	1.847 (1.652-2.043)
Farmers' markets selling animal products (%)	64.91	64.75	66.57	0.861 (0.666-1.056)
Population within half-mile of park (%)	11.45	11.54	10.54	-0.874 (-0.937--0.811)
Private Insurance coverage (%)	53.58	53.69	52.37	-1.484 (-1.581--1.387)
Medicaid coverage (%)	22.21	22.15	22.87	1.446 (1.367-1.526)
Medicare coverage (%)	20.52	20.44	21.38	0.440 (0.390-0.491)
Median household income, ZIP code	50,299.73	50,427.88	48,963.02	-2,422.707(-2,559.644--2,285.770)
**Medication of lipid disorders**				
Simvastatin	14,349	9,721 (1.57%)	4,628 (7.70%)	2.420 (2.328-2.515)
Atorvastatin calcium	13,713	9,106 (1.47%)	4,607 (7.67%)	2.767 (2.661-2.878)
Pravastatin sodium	4,310	2,965 (0.48%)	1,345 (2.24%)	2.400 (2.238-2.572)
Rosuvastatin calcium	3,938	2,509 (0.40%)	1,429 (2.38%)	3.166 (2.952-3.395)
Lovastatin	2,933	1,998 (0.32%)	935 (1.56%)	2.129 (1.958-2.314)
Fenofibrate nanocrystallized	1,054	680 (0.11%)	374 (0.62%)	4.470 (3.898-5.117)
**Medication of Type 2 diabetes**				
Metformin hcl	10,734	6,502 (1.05%)	4,232 (7.05%)	5.013 (4.802-5.233)
Blood sugar diagnostic	8,031	5,411 (0.87%)	2,620 (4.36%)	4.334 (4.114-4.564)
Lancets	4,036	2,761 (0.44%)	1,275 (2.12%)	4.507 (4.187-4.849)
Glipizide	3,143	1,731 (0.28%)	1,412 (2.35%)	4.176 (3.867-4.510)
Insulin aspart	2,083	1,483 (0.24%)	600 (1.00%)	3.836 (3.453-4.256)
Insulin lispro	1,913	1,358 (0.22%)	555 (0.92%)	4.345 (3.897-4.839)
Glyburide	1,349	798 (0.13%)	551 (0.92%)	4.957 (4.399-5.582)
Sitagliptin phosphate	1,246	690 (0.11%)	556 (0.93%)	4.793 (4.243-5.412)
Insulin detemir	1,037	632 (0.10%)	405 (0.67%)	4.164 (3.631-4.770)
**Medication of cardiovascular diseases**				
Lisinopril	15,397	7,687 (1.24%)	7,710 (12.84%)	6.810 (6.573-7.056)
Hydrochlorothiazide	8,105	3,994 (0.64%)	4,111 (6.84%)	6.592 (6.282-6.918)
Metoprolol tartrate	7,775	4,298 (0.69%)	3,477 (5.79%)	3.747 (3.566-3.937)
Metoprolol succinate	6,735	3,802 (0.61%)	2,933 (4.88%)	3.702 (3.511-3.904)

Amlodipine besylate	6,467	3,068 (0.49%)	3,399 (5.66%)	5.152 (4.883-5.436)
Atenolol	5,750	3,026 (0.49%)	2,724 (4.54%)	4.762 (4.498-5.041)
Propranolol hcl	4,318	3,548 (0.57%)	770 (1.28%)	2.638 (2.424-2.867)
Losartan potassium	3,667	1,760 (0.28%)	1,907 (3.17%)	5.085 (4.739-5.456)
Diltiazem hcl	2,251	1,321 (0.21%)	930 (1.55%)	2.726 (2.489-2.984)
Enalapril maleate	1,109	568 (0.09%)	541 (0.90%)	5.223 (4.591-5.940)
Triamterene or hydrochlorothiazid	1,067	572 (0.09%)	495 (0.82%)	4.234 (3.715-4.823)
Digoxin	993	653 (0.11%)	340 (0.57%)	1.526 (1.328-1.751)
Verapamil hcl	990	630 (0.10%)	360 (0.60%)	3.758 (3.254-4.334)
Valsartan	796	388 (0.06%)	408 (0.68%)	4.115 (3.547-4.775)

[a]COPD: chronic obstructive pulmonary disease.
