# Supplementary material for: Prediction of Incident Hypertension Within the Next Year: Prospective Study Using Statewide Electronic Health Records and Machine Learning
Source: J Med Internet Res. 2018 Jan 30;20(1):e22. doi: 10.2196/jmir.9268 (PMC5811646; doi:10.2196/jmir.9268)
Supplement: Multimedia Appendix 4 [file jmir_v20i1e22_app4.pdf]

Appendix 4. Distribution of impactful risk predictors across the five risk categories, specified as features of demographics, diagnosed diseases, and clinical utilization

Risk category		Very low	Low	Medium	High	Very high
Risk score intervals		[0, 0.05]	[0.05, 0.1]	[0.1, 0.2]	[0.2, 0.4]	[0.4, 1]
Population		381,544	104,565	99,415	53,957	41,329
Demographic features						
Age	<35	89.27%	44.45%	9.68%	6.69%	8.22%
	35-49	6.71%	51.04%	54.11%	26.81%	27.42%
	50-64	2.51%	3.19%	27.71%	34.57%	30.45%
	≥65	1.49%	1.25%	8.45%	31.90%	33.88%
Gender	Female	57.87%	63.00%	54.70%	46.89%	48.41%
Diagnosed diseases						
Prehypertension		0.02%	0.13%	0.28%	1.32%	1.59%
Cardiovascular diseases		0.65%	1.94%	4.21%	13.35%	25.08%
Type 2 diabetes		0.26%	0.96%	2.34%	9.86%	20.17%
Chronic nephritis		0.02%	0.08%	0.26%	0.81%	2.08%
Chronic kidney disease		0.01%	0.07%	0.22%	0.74%	1.60%
Idiopathic hypersomnia		0.09%	0.46%	0.77%	1.40%	1.62%
Acquired hemolytic anemia		0.43%	1.06%	1.43%	2.02%	2.54%
Hyposmolality or hyponatremia		0.01%	0.04%	0.08%	0.19%	0.32%
Hypopotassemia		0.03%	0.10%	0.17%	0.32%	0.48%
Disorders of lipid metabolism		0.27%	3.08%	8.03%	11.51%	8.76%
Hyperlipidemia		0.16%	1.98%	5.39%	8.09%	6.21%
Mixed hyperlipidemia		0.02%	0.25%	0.64%	0.92%	0.69%
Pure hypercholesterolem		0.07%	0.73%	1.88%	2.48%	1.81%
Liver disorders		0.20%	0.76%	1.13%	1.38%	1.31%
COPD[a]		0.43%	1.11%	2.24%	3.73%	3.61%
Clinical utilization						

Inpatient admissions last year	0.04	0.06	0.07	0.1	0.13
Outpatient visits last year	0.85	0.92	0.95	0.96	0.97
Patient's estimated cost last year (US $)	1397.23	2030.07	2350.75	2960.41	3630.37
Number of chronic diseases	2.30	2.38	2.71	3.19	3.56
Number of different medications	6.33	7.07	7.48	8.55	12.12
Number of medications	18.69	21.92	23.71	27.76	41.35
Number of abnormal lab tests	0.13	0.25	0.30	0.36	0.40
Number of lab tests	0.18	0.32	0.37	0.42	0.45

[a]COPD: chronic obstructive pulmonary disease.
