# Supplementary material for: Prediction of Incident Hypertension Within the Next Year: Prospective Study Using Statewide Electronic Health Records and Machine Learning
Source: J Med Internet Res. 2018 Jan 30;20(1):e22. doi: 10.2196/jmir.9268 (PMC5811646; doi:10.2196/jmir.9268)
Supplement: Multimedia Appendix 5 [file jmir_v20i1e22_app5.pdf]

Appendix 5. ROC curves and AUC values of subgroups

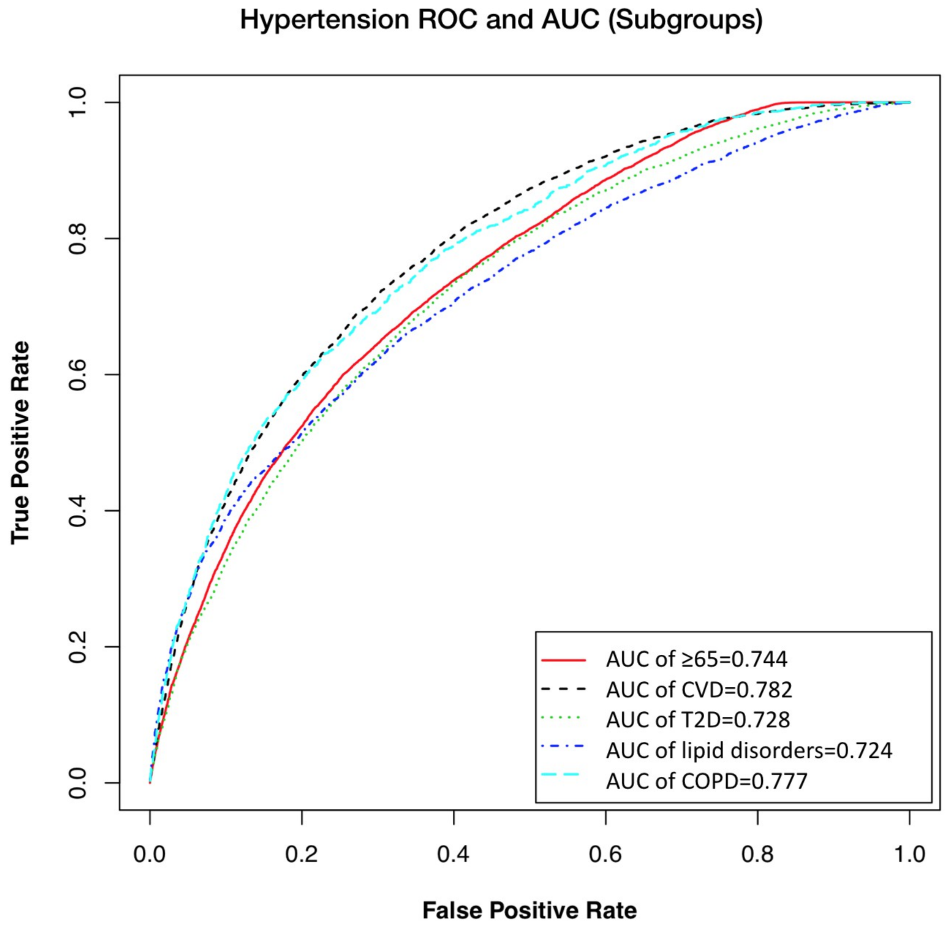

Figure Appendix 4 ROC curves and AUC values of the different population subgroups, including people ≥65 years old, and those having diagnoses of cardiovascular disease (CVD), type 2 diabetes (T2D), lipid disorders or chronic obstructive pulmonary disease (COPD), respectively.
