# Supplementary material for: Prediction of Incident Hypertension Within the Next Year: Prospective Study Using Statewide Electronic Health Records and Machine Learning
Source: J Med Internet Res. 2018 Jan 30;20(1):e22. doi: 10.2196/jmir.9268 (PMC5811646; doi:10.2196/jmir.9268)
Supplement: Multimedia Appendix 6 [file jmir_v20i1e22_app6.pdf]

Appendix 6. Distribution of impactful medications of depression, anxiety, and schizophrenia across the five risk categories

Risk category	Very low	Low	Medium	High	Very high
Intervals	[0, 0.05]	[0.05, 0.1]	[0.1, 0.2]	[0.2, 0.4]	[0.4, 1]
Case	381,544	104,565	99,415	53,957	41,329
Depression Medications					
Mirtazapine	0.30%	0.66%	0.83%	1.20%	1.58%
Nortriptyline	0.12%	0.36%	0.42%	0.52%	0.74%
Paroxetine	0.39%	1.03%	1.00%	1.37%	2.12%
Sertraline	2.32%	3.11%	2.91%	3.62%	5.77%
Trazodone	1.01%	2.35%	2.65%	3.25%	4.58%
Bupropion	1.32%	3.11%	2.65%	2.74%	4.29%
Citalopram hydrobromide	1.05%	2.02%	1.75%	2.16%	3.44%
Escitalopram oxalate	0.95%	1.72%	1.62%	1.70%	2.57%
Amitriptyline	0.60%	1.49%	1.54%	1.63%	2.82%
Anxiety Medications					
Buspirone	0.93%	2.63%	2.82%	3.46%	4.97%
Lorazepam	0.44%	0.81%	0.76%	0.78%	1.19%
Diazepam	0.67%	1.95%	1.92%	2.08%	2.96%
Alprazolam	0.53%	1.72%	1.78%	2.25%	3.43%
Escitalopram oxalate	0.95%	1.72%	1.62%	1.70%	2.57%
Schizophrenia Medications					
Clozapine	0.02%	0.09%	0.10%	0.13%	0.16%
Aripiprazole	0.59%	0.80%	0.81%	0.77%	0.91%
Olanzapine	0.15%	0.39%	0.43%	0.52%	0.59%
Quetiapine fumarate	0.38%	0.77%	0.78%	0.85%	1.05%
Ziprasidone	0.08%	0.18%	0.17%	0.19%	0.25%
