# Supplementary material for: Prediction of Incident Hypertension Within the Next Year: Prospective Study Using Statewide Electronic Health Records and Machine Learning
Source: J Med Internet Res. 2018 Jan 30;20(1):e22. doi: 10.2196/jmir.9268 (PMC5811646; doi:10.2196/jmir.9268)
Supplement: Multimedia Appendix 7 [file jmir_v20i1e22_app7.pdf]

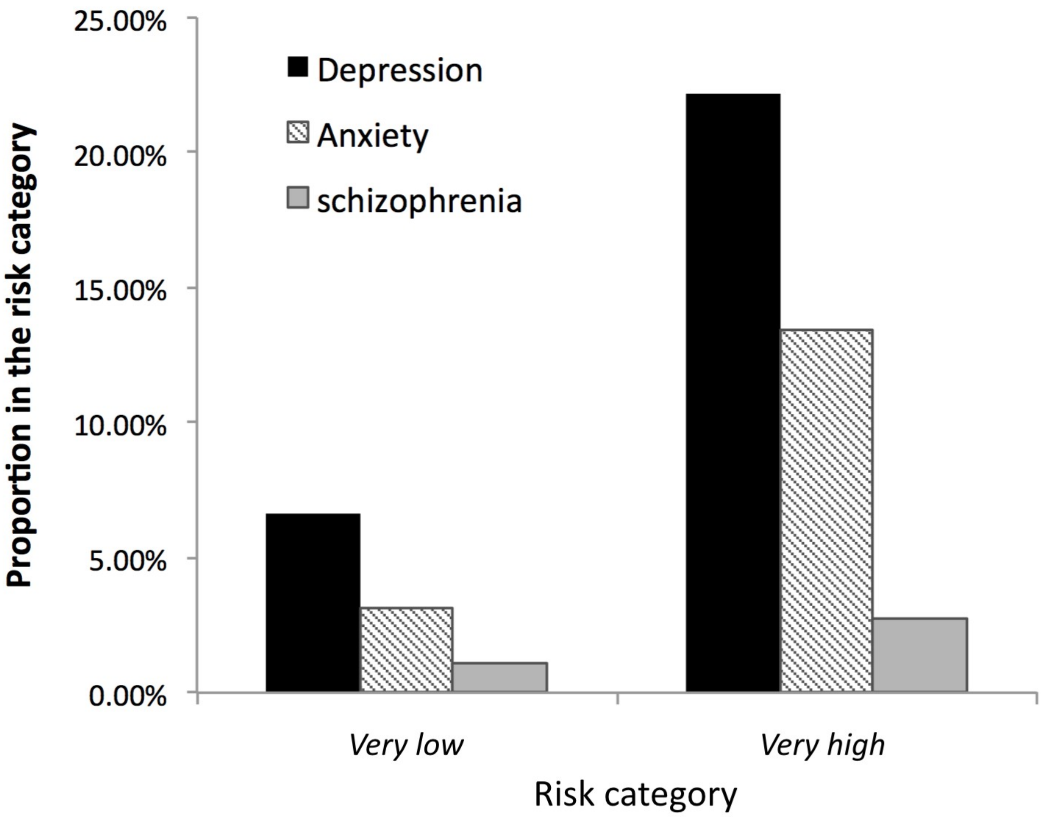

Figure Appendix 7. Constituent ratios of three mental diseases (depression, anxiety, and schizophrenia) in the *very low risk* and *very high risk* categories
