# Supplementary material for: Prediction of Incident Hypertension Within the Next Year: Prospective Study Using Statewide Electronic Health Records and Machine Learning
Source: J Med Internet Res. 2018 Jan 30;20(1):e22. doi: 10.2196/jmir.9268 (PMC5811646; doi:10.2196/jmir.9268)
Supplement: Multimedia Appendix 8 [file jmir_v20i1e22_app8.pdf]

Appendix 8. Distribution of social determinant indicators across the five categories with *very low*, *low*, *medium*, *high*, and *very high* risk of hypertension

Risk category	Very low	Low	Medium	High	Very high
Intervals	[0, 0.05]	[0.05, 0.1]	[0.1, 0.2]	[0.2, 0.4]	[0.4, 1]
Case	381,544	104,565	99,415	53,957	41,329
Low-educated population (%)	8.73	8.75	8.95	9.05	9.20
High-educated population (%)	49.05	48.81	48.22	47.97	47.85
Median household income, ZIP code	51040.43	50294.47	49038.51	48577.47	48662.88
Low income and low access to store[a] (%)	3.85	3.81	3.73	3.68	3.69
Grocery stores/1000 pop[b]	0.26	0.27	0.27	0.28	0.27
Convenience stores/1000 pop	0.67	0.68	0.70	0.71	0.70
Farmers' markets selling fruit and vegetables[c] (%)	60.96	60.31	61.54	63.18	64.00
Farmers' markets selling animal products (%)	64.76	63.85	64.91	66.39	67.07
County population within half-mile of park (%)	11.92	11.35	10.71	10.37	10.46
Private Insurance coverage (%)	54.15	53.77	52.65	51.94	52.09
Medicaid coverage (%)	21.97	22.12	22.61	22.83	22.97
Medicare coverage (%)	20.13	20.42	21.10	21.70	21.58

[a] Percentage of people in a county with low income and living more than 1 mile from a supermarket or large grocery store if in an urban area, or more than 10 miles from a supermarket or large grocery store if in a rural area. [b] The number of supermarkets and grocery stores in the county per 1,000 county residents. [c] Percentage of farmers' markets in the county that sell fresh fruits and vegetables
